# Integration of SARS-CoV-2 RNA in infected human cells by retrotransposons: an unlikely hypothesis and old viral relationships

**DOI:** 10.1186/s12977-021-00578-w

**Published:** 2021-10-29

**Authors:** Nicole Grandi, Enzo Tramontano, Ben Berkhout

**Affiliations:** 1 Laboratory of Molecular Virology, Department of Life and Environmental Sciences, University of Cagliari – Cittadella Universitaria di Monserrato, SS554 Monserrato (Cagliari), Italy; 2 Istituto di Ricerca Genetica e Biomedica (IRGB), CNR – SS554, Monserrato (Cagliari), Italy; 3 Laboratory of Experimental Virology, Department of Medical Microbiology, Amsterdam Medical Centers, Meibergdreef 15, 1105 AZ Amsterdam, The Netherlands

**Keywords:** SARS-CoV-2, COVID19, LINE, L1, Retrotransposition, HERV, HERV-W, Chimeric RNA

## Abstract

Zhang et al. (Proc Natl Acad Sci 118:e2105968118, 2021) recently reported that SARS-CoV-2 RNA can be retrotranscribed and integrated into the DNA of human cells by the L1 retrotransposon machinery. This phenomenon could cause persistence of viral sequences in patients and may explain the prolonged PCR-positivity of SARS-CoV-2 infected patients, even long after the phase of active virus replication has ended. This commentary does critically review the available data on this topic and discusses them in the context of findings made for other exogenous viruses and ancestral endogenous retroviral elements.

## Text body

The COVID pandemic that started at the end of 2019 led to a remarkable mobilization of scientific efforts as evidenced by > 175.000 publications to date. Among these, the work by Zhang et al. triggered an animated debate in the scientific community [1]. Based on studies performed in cultured cells transfected with DNA encoding the retrotransposon L1 (long interspersed nuclear elements 1), authors proposed that SARS-CoV-2 RNA, in particular the subgenomic RNA encoding the nucleocapsid (NC), can be converted into dsDNA and integrated into the cellular genome by the L1 retrotransposition machinery [1]. These SARS-CoV-2 sequences can be expressed in patients as chimeric cellular-viral transcripts, which could explain the long-term PCR positivity for viral RNA in patients who recovered from COVID. A similar hypothesis was proposed by Yin and co-workers who observed that infection by SARS-CoV-2 (as well as other human coronaviruses) causes upregulation of retrotransposon expression, leading to the formation of chimeric virus-retrotransposon transcripts [2].

These original reports opened a heated debate on the correctness of the findings and their relevance for recovered COVID patients and subsequent work was initiated to test alternative explanations. It was proposed early on that the observed chimeric RNAs could be artifacts generated during cDNA library preparation. Two findings hint at this possibility. First, the directionality of the observed chimeric transcripts, in which a large fraction of SARS-CoV-2 RNA derives from the (–) strand, in contrast to the predominance of ( +) strand RNAs in SARS-CoV-2 natural infection. Second, the absence of the 3’ end and polyA tail of the viral genome, which are commonly present in integrated sequences processed by L1 elements.

The origin of the chimeric human-SARS-CoV-2 reads in RNA-seq libraries was subsequently investigated in a dedicated study [3], which showed that such hybrid sequences arose also between SARS-CoV-2 RNA and transcripts encoded by mitochondrial DNA or episomal adenoviral DNA in transfected cells, thus being unlikely the result of genuine SARS-CoV-2 integration. Other studies focused on detecting SARS-CoV-2 retrotransposition events in deep sequencing data, confirming the absence of genuine L1-mediated integration events and suggesting that the observed chimeric transcripts had emerged during RNA-seq library construction [4–7]. Importantly, such chimeric reads were also identified when RNA from infected human cells was mixed before library preparation with RNA from uninfected or unrelated vertebrate cells [4, 7]. In addition, the lack of reproducibility of the observed host-virus chimeric transcripts across SARS-CoV-2 patient samples corroborated the idea that these sequences arose from stochastic, artifactual events at the RNA-seq level (e.g. random ligations, template switching and/or sequence alignment errors). Consistent with this notion, the chimeric reads are mostly composed of abundantly expressed cellular and viral transcripts. Together, the collective evidence for genuine SARS-CoV-2 DNA formation and integration remains sparse [7].

To put these recent reports in a broader context, some consideration should be given to the molecular biology of L1 elements and their interplay with viruses. L1 elements represent the most abundant subfamily of non-LTR retrotransposons, accounting for ∼17% of the human genome. L1 elements are autonomous for self-mobilization by encoding two proteins (ORF1 and ORF2) that together mediate reverse transcription of their own RNA and subsequent integration of the resulting dsDNA in the cellular genome [8]. This process shows some cross-activity on non-autonomous retrotransposons. Despite the accumulation of inactivating mutations, a subset of 80–100 L1 elements remains active in the human genome. Accordingly, L1 retrotransposition has been observed at early stages of embryonic development, and > 100 de novo L1 insertions have been linked to heritable genetic disorders [9]. Beyond the germ line and pluripotent stem cells, L1 activity has been reported at the somatic level in neuronal progenitors and various human tumors, possibly being responsible for mutagenic events [9]. For these reasons, L1 elements are intensively being studied in diverse diseases and they were reported to be upregulated in different pathologies and especially cancer. However, there is no direct evidence for retrotransposition as a cause of disease. This also holds true for the multi-step process of tumorigenesis, where putative LINE contributions could be due to indirect effects, e.g. by non-specific epigenetic changes in cancer cells.

There are few reports on L1-mediated mobilization of viral transcripts. In hepatocellular carcinoma (HCC) induced by hepatitis B virus (HBV), recurrent integration of HBV subgenomic RNAs was reported to yield a chimeric long non-coding RNA between the HBV mRNA for the X antigen (HBx) and L1 RNA in > 23% of patient samples [10]. Of note, this HBx-L1 chimeric RNA is reported to promote malignant transformation and hepatic injury [11]. Unlike for retroviruses, integration is not a mandatory step in the HBV replication cycle and the mechanism of HBV integration in HCC cells remains poorly characterized. The observation that ∼ 90% of HBV-induced HCC cells contain at least one integrated HBV-DNA fragment, combined with their preferential localization in or near repetitive elements, could cautiously suggest a possible role of L1 elements in the mobilization of short HBV transcripts [12]. This scenario is consistent with the fact that HBV replication occurs in the nucleus and is corroborated by the presence of HBV-integrations in most HCC samples, whose abundance seems to negatively correlate with patient survival [12]. Of note, ∼ 40% of viral breakpoints observed upon HBV integration are restricted to an 1800-bp genome portion including the viral enhancer, X gene and core gene, which may contain features that are recognized by the L1 machinery. Perhaps a coincidence, but the size of the above mobilized HBV genome portion is comparable to that of the mobilized SARS-CoV-2 RNA fragment (1,662 bp) reported by Zhang et al. [1]. Specific breaks in the viral genome also occur during SV40-BK virus oncogenesis, leading to upregulated expression of the viral oncogene. It is important to stress that integration as detected in tumor cells does NOT occur during normal virus replication.

The most remarkable case of L1-virus interplay does however not involve “modern” human viruses, but rather a group of human endogenous retroviruses (HERVs) that were acquired by the primate genome some 20–43 million years ago through infection of the germ line by now-extinct retroviruses [13]. The hallmark of all retroviruses is reverse transcription of their RNA genome into dsDNA that integrates in the genome of the infected cells. Hence, germline integration of these ancestral retroviruses allowed their inheritance as Mendelian genes and vertical transmission to the offspring. HERV retrotransposons currently constitute ∼ 8% of our genome and have occasionally been used to develop novel and important physiological processes like placenta formation [14, 15]. The HERV-W group is unique for its colonization dynamics: among the 213 members, 135 (63%) are not direct retroviral integrations, but rather processed pseudogenes that were generated through mobilization of HERV-W transcripts by the L1 machinery [13, 16]. Only this HERV group shows such L1-dependency, although the determinants for the specific interaction with L1 remain unclear. Sequence analyses indicated that mobilization is 2.5-fold more efficient for subgroup 1 HERV-W members, suggesting the presence of preferential sequence signatures for L1 recognition [13]. Besides retroviruses, which have reverse transcription and integration as a stable biological feature, an example of human endogenous viral elements (EVEs) that have likely involved L1 in their formation are the bornavirus-like elements, i.e. the only non-retroviral RNA virus-derived EVEs [17]. This scenario is supported by the fact that most of such elements originate from reverse-transcription and integration of the mRNA coding for ancient bornavirus nucleoprotein, with genomic localization and flanking sequences being consistent with L1 action [18].

## Conclusions

Overall, it seems unlikely that the L1 machinery is responsible for “genuine” SARS-CoV-2 genomic integration in infected cells. In fact, this process of *trans*-mobilization has been infrequent even for exogenous retroviruses that use integration as a key feature of their replication cycle. Accordingly, despite the evidence that the primate DNA genome has been invaded repeatedly by exogenous retroviral infections during evolution, only a single HERV group was copied-and-pasted by the L1 machinery, and the molecular signatures that facilitate viral RNA retrotransposition by the L1 apparatus are still poorly defined. It is known that SINE non-autonomous retrotransposons exploit the L1 machinery for retrotransposition, which is based on 3′ end sequence similarity between LINEs and SINEs [8]. Given the nucleotide sequence diversity of L1 3’ ends and the candidate viral sequences that were mobilized by L1 (e.g. HERV-W pseudogenes and HBx mRNA), it seems likely that retrotransposition involved the recognition of RNA secondary structures and other spatial features instead of a specific sequence. The cell type in which integration occurs may also have a considerable impact. Whereas HERV-W pseudogenes are formed in germ line cells, which are known to have high L1 physiological activity, HBV and possibly SARS-CoV-2 integrations were described in somatic cells. This still-unveiled selectivity of L1 mobilization towards certain viral transcripts, in addition to the concrete possibility that chimeric SARS-CoV-2/cellular RNAs can artefactually arise during the amplification/sequencing procedures, remain major confounding factors in the characterization of putative de novo retrotransposition events of SARS-CoV-2 mRNA.

Further studies are necessary to assess the actual impact of active retrotransposons in the mobilization of viral and host transcripts and to characterize the molecular mechanisms underlying their integration in the host genome and their subsequent expression.

## Data Availability

Not applicable.

## Abbreviations

NC: Nucleocapsid
L1: Long interspersed nuclear elements 1
HCC: Hepatocellular carcinoma
HBV: Hepatitis B virus
HBx: Hepatitis B virus X antigen
HERVs: Human endogenous retroviruses
EVEs: Endogenous viral elements

## References

[1] Zhang L, Richards A, Barrasa MI, Hughes SH, Young RA, Jaenisch R (2021). Reverse-transcribed SARS-CoV-2 RNA can integrate into the genome of cultured human cells and can be expressed in patient-derived tissues. Proc Natl Acad Sci.

[2] Yin Y, Liu XZ, He X, Zhou LQ (2021). Exogenous coronavirus interacts with endogenous retrotransposon in human cells. Front Cell Infect Microbiol.

[3] Kazachenka A, Kassiotis G (2021). SARS-CoV-2-host chimeric RNA-sequencing reads do not necessarily arise from virus integration into the host DNA. Front Microbiol.

[4] Chen Y-S, Lu S, Zhang B, Du T, Li W-J, Lei M (2021). Comprehensive analysis of RNA-seq and whole genome sequencing data reveals no evidence for SARS-CoV-2 integrating into host genome. Protein Cell.

[5] Parry R, Gifford RJ, Lytras S, Ray SC, Coin LJM (2021). No evidence of SARS-CoV-2 reverse transcription and integration as the origin of chimeric transcripts in patient tissues. Proc Natl Acad Sci.

[6] Smits N, Rasmussen J, Bodea GO, Amarilla AA, Gerdes P, Sanchez-Luque FJ (2021). No evidence of human genome integration of SARS-CoV-2 found by long-read DNA sequencing. Cell Rep.

[7] Yan B, Chakravorty S, Mirabelli C, Wang L, Trujillo-Ochoa JL, Chauss D (2021). Host-virus chimeric events in SARS-CoV-2-infected cells are infrequent and artifactual. J Virol.

[8] Ferrari R, Grandi N, Tramontano E, Dieci G (2021). Retrotransposons as drivers of mammalian brain evolution. Life.

[9] Suarez NA, Macia A, Muotri AR (2018). LINE-1 retrotransposons in healthy and diseased human brain. Dev Neurobiol.

[10] Lau CC, Sun T, Ching AKK, He M, Li JW, Wong AM (2014). Viral-human chimeric transcript predisposes risk to liver cancer development and progression. Cancer Cell.

[11] Liang HW, Wang N, Wang Y, Wang F, Fu Z, Yan X (2016). Hepatitis B virus-human chimeric transcript HBx-LINE1 promotes hepatic injury via sequestering cellular microRNA-122. J Hepatol.

[12] Sung WK, Zheng H, Li S, Chen R, Liu X, Li Y (2012). Genome-wide survey of recurrent HBV integration in hepatocellular carcinoma. Nat Genet.

[13] Grandi N, Cadeddu M, Blomberg J, Tramontano E (2016). Contribution of type W human endogenous retroviruses to the human genome: characterization of HERV-W proviral insertions and processed pseudogenes. Retrovirology.

[14] Grandi N, Tramontano E (2018). HERV envelope proteins: Physiological role and pathogenic potential in cancer and autoimmunity. Front Microbiol.

[15] Grandi N, Tramontano E (2018). Human endogenous retroviruses are ancient acquired elements still shaping innate immune responses. Front Immunol.

[16] Pavlícek A, Paces J, Elleder D (2002). Processed pseudogenes of human endogenous retroviruses generated by LINEs: their integration, stability, and distribution. Genome Res.

[17] Honda T (2017). Potential links between hepadnavirus and bornavirus sequences in the host genome and cancer. Front Microbiol.

[18] Horie M, Honda T, Suzuki Y, Kobayashi Y, Daito T, Oshida T (2010). Endogenous non-retroviral RNA virus elements in mammalian genomes. Nature.

